# Differential Neutralizing Activities of a Single Domain Camelid Antibody (V_H_H) Specific for Ricin Toxin’s Binding Subunit (RTB)

**DOI:** 10.1371/journal.pone.0099788

**Published:** 2014-06-11

**Authors:** Cristina Herrera, David J. Vance, Leslie E. Eisele, Charles B. Shoemaker, Nicholas J. Mantis

**Affiliations:** 1 Division of Infectious Disease, Wadsworth Center, New York State Department of Health, Albany, New York, United States of America; 2 Department of Biomedical Sciences, University at Albany School of Public Health, Albany, New York, United States of America; 3 Scientific Cores, Wadsworth Center, New York State Department of Health, Albany, New York, United States of America; 4 Department of Infectious Disease and Global Health, Tufts Cummings School of Veterinary Medicine, North Grafton, Massachusetts, United States of America; Naval Research Laboratory, United States of America

## Abstract

Ricin, a member of the A-B family of ribosome-inactivating proteins, is classified as a Select Toxin by the Centers for Disease Control and Prevention because of its potential use as a biothreat agent. In an effort to engineer therapeutics for ricin, we recently produced a collection of alpaca-derived, heavy-chain only antibody V_H_ domains (V_H_H or “nanobody”) specific for ricin’s enzymatic (RTA) and binding (RTB) subunits. We reported that one particular RTB-specific V_H_H, RTB-B7, when covalently linked via a peptide spacer to different RTA-specific V_H_Hs, resulted in heterodimers like V_H_H D10/B7 that were capable of passively protecting mice against a lethal dose challenge with ricin. However, RTB-B7 itself, when mixed with ricin at a 1∶10 toxin:antibody ratio did not afford any protection *in vivo*, even though it had demonstrable toxin-neutralizing activity *in vitro*. To better define the specific attributes of antibodies associated with ricin neutralization *in vitro* and *in vivo*, we undertook a more thorough characterization of RTB-B7. We report that RTB-B7, even at 100-fold molar excess (toxin:antibody) was unable to alter the toxicity of ricin in a mouse model. On the other hand, in two well-established cytotoxicity assays, RTB-B7 neutralized ricin with a 50% inhibitory concentration (IC_50_) that was equivalent to that of 24B11, a well-characterized and potent RTB-specific murine monoclonal antibody. In fact, RTB-B7 and 24B11 were virtually identical when compared across a series of *in vitro* assays, including adherence to and neutralization of ricin after the toxin was pre-bound to cell surface receptors. RTB-B7 differed from both 24B11 and V_H_H D10/B7 in that it was relatively less effective at blocking ricin attachment to receptors on host cells and was not able to form high molecular weight toxin:antibody complexes in solution. Whether either of these activities is important in ricin toxin neutralizing activity *in vivo* remains to be determined.

## Introduction

There are ongoing initiatives to develop countermeasures against ricin, a Select Toxin, as classified by the Centers for Disease Control and Prevention (CDC), and which has been the subject of a number of recent high profile bioterrorism incidents in the United States [1], [2]. Ricin is a glycoprotein derived from the castor bean plant, *Ricinus communis*, and a member of the medically important family of A-B toxins [3]. Ricin’s enzymatic subunit (RTA) is an RNA N-glycosidase that inactivates eukaryotic ribosomes by catalyzing the hydrolysis of a universally conserved residue within the so-called sarcin/ricin loop (SRL) of 28S rRNA [4], [5]. Ricin’s B subunit (RTB) is a galactose- and N-acetylgalactosamine (Gal/GalNAc)-specific lectin that has two important functions in cytotoxicity. First, RTB promotes ricin attachment and endocytosis of ricin into all mammalian cell types, including epithelial cells, sinusoidal endothelial cells, and macrophages [6], [7]. Second, following endocytosis, RTB mediates the retrograde transport of RTA from the plasma membrane to the trans-Golgi network (TGN) and endoplasmic reticulum (ER), where RTA is liberated from RTB and retro-translocated into the cell cytoplasm [8], [9].

**Figure 1:**
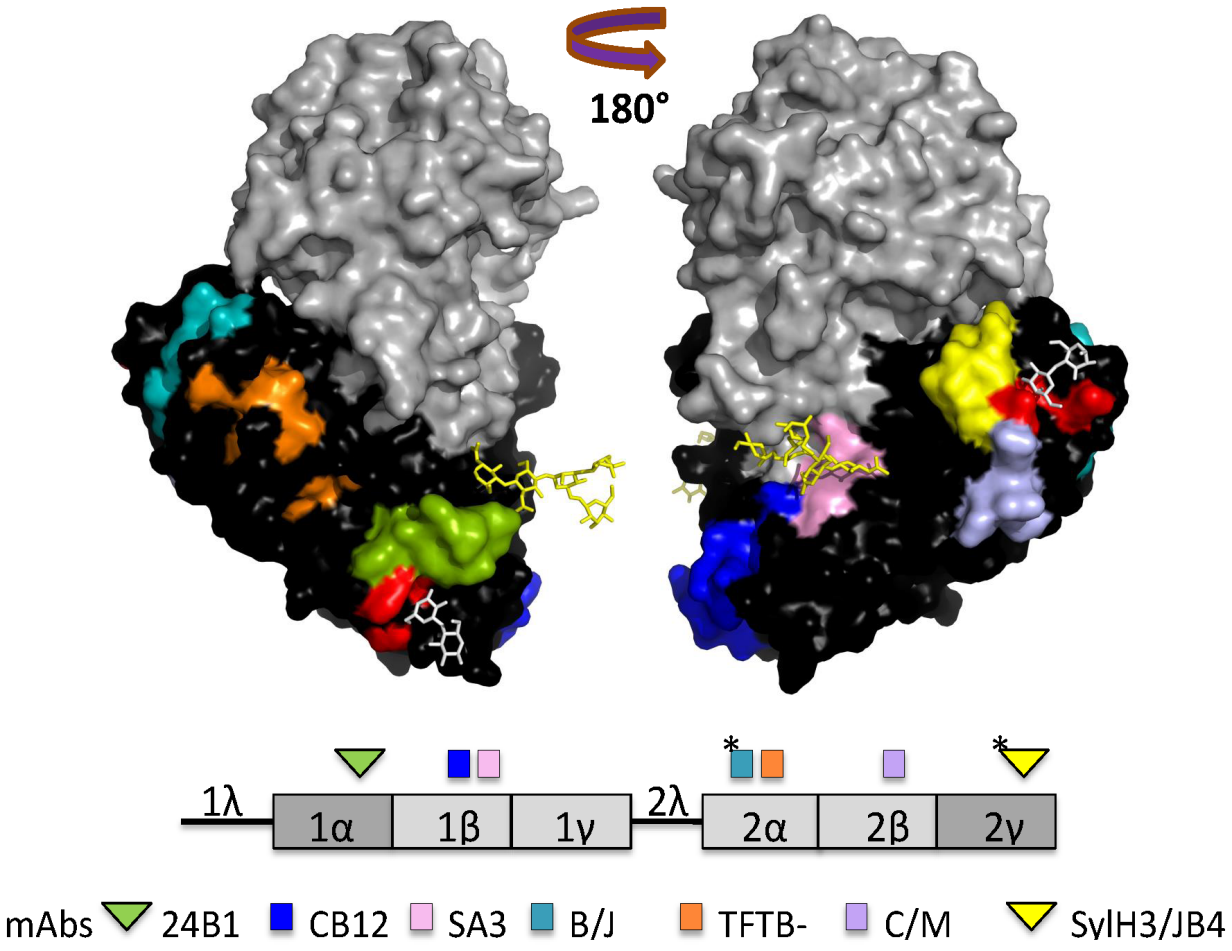
Previously identified and characterized epitopes on RTB recognized by neutralizing and non-neutralizing mAbs. X-ray crystal structure of ricin holotoxin visualized using PyMOL and based on PDB file 2AAI [55]. RTA (grey), RTB (black), ricin’s N-linked mannose side chains (yellow sticks) and lactose moieties (white sticks) are shown in the upper panel. Confirmed and putative epitopes (*) recognized by neutralizing (triangles) and non-neutralizing (squares) RTB-specific mAbs are color-coded on the holotoxin structure to match RTB’s linear subdomain organization in the lower panel. This figure was modified from an earlier version [26].

Considering its essential role in toxin uptake and trafficking, RTB is an appealing target for antibody-based therapeutics. Structurally, RTB is composed of two globular domains (1 and 2) each containing three homologous sub-domains (α, β and γ), although only the external sub-domains (1α and 2γ) retain functional carbohydrate recognition activity (**Figure 1**) [6], [10]. Sub-domain 1α (residues 17–59) is Gal-specific and is considered a “low affinity” carbohydrate recognition domain (CRD), whereas sub-domain 2γ (residues 228–262) binds both Gal and GalNAc and is considered a “high affinity” CRD [11]–[13]. RTB has four intramolecular disulfide bonds, in addition to the single intermolecular disulfide bond that joins it to RTA [14], [15]. Finally, RTB has two N-linked mannose side chains that have been postulated to interact with mannose-binding protein(s) during ricin intracellular transport and/or influence the intracellular stability of RTB [16]–[19].

A number of RTB-specific murine monoclonal antibodies (mAbs) have been described in the literature, although only a handful have been shown to have toxin-neutralizing activity [20]–[26]. The two most well characterized mAbs from our laboratory are SylH3 and 24B11. Although SylH3 and 24B11 are each able to neutralize ricin *in vitro* and *in vivo*, they apparently do so by different mechanisms [27]–[29]. SylH3 virtually abolishes ricin’s ability to adhere to host cell receptors, and is therefore postulated to neutralize ricin by steric hindrance. 24B11 also affects ricin binding to host cell surfaces, although this activity alone does not fully account for 24B11’s potent toxin-neutralizing activity. Rather, we recently reported that 24B11 is able to associate with ricin in solution or after the toxin has adhered cell surface receptors. Surface-bound 24B11 is subsequently endocytosed as a toxin:antibody complex and interferes with retrograde transport of ricin to the TGN [27]. We have also reported that SylH3 and 24B11 Fab fragments were as effective as the full length IgGs at neutralizing ricin *in vitro* and *in vivo*, indicating that in the case of SylH3 and 24B11, neither bivalency nor Fc-domains are necessary determinants of toxin-neutralizing activity [28].

However, the relationships between affinity, avidity, epitope specificity, Fc-mediated effector functions, and toxin-neutralizing activity *in vitro* and *in vivo* remains poorly understood in the case of ricin. Defining mechanisms of toxin-neutralizing activity by RTB-specific antibodies has been particularly challenging because only a limited number of conventional murine mAbs besides 24B11 and SylH3 are available [21], [23], [25]–[27], [29]. Moreover, conventional mAbs like 24B11 and SylH3 are not easily reengineered or modified to permit a systematic analysis of the factors that render an antibody effective at neutralizing ricin. Such versatility can only be achieved with single-domain camelid-derived antibodies, referred to as V_H_Hs or simply “nanobodies”, which are small, generally highly stable, and easily expressed in *Escherichia coli* or on the surface of filamentous bacteriophages like M13 [30]. For example, RTA- and RTB-specific single chain nanobodies were affinity isolated from a phage-displayed semisynthetic llama library and have proven useful for a number of diagnostic applications [31]–[34]. Camelid-derived, single domain antibodies against Shiga toxin, botulinum neurotoxins (BoNT) and *Clostridium difficile* toxins have also been described [35]–[38].

We recently produced and partially characterized a collection of ricin-specific V_H_Hs from alpacas [39]. We identified 11 unique RTA-specific V_H_Hs and 9 unique RTB-specific V_H_Hs. Among the nine unique RTB-specific V_H_Hs, only one, RTB-B7, had demonstrable toxin-neutralizing activity (TNA) in a Vero cell cytotoxicity assay, although a number of others like RTB-D12 had apparent affinities for ricin that were equal to or less than RTB-B7’s [39]. RTB-B7 was covalently linked via a short peptide spacer (GGGGS)_3_ to three different RTA-specific V_H_Hs, including RTA-D10, resulting in three different V_H_H “heterodimers” that each proved capable of passively protecting mice against a lethal dose ricin challenge. We have subsequently characterized two of the three RTA-specific V_H_H components of the three heterodimers in great detail, including solving the X-ray crystal structures of the V_H_H monomers in complex with RTA (MJ Rudolph, DJ Vance, J Cheung, MC Franklin, F Burshteyn, MS Cassidy, EN Gary, C Herrera, CB Shoemaker, and NJ Mantis, *manuscript in revision*). However, very little is known about RTB-B7. It is not known if RTB-B7, at high doses, is able to passively protect mice from ricin toxin, or how RTB-B7 compares to RTB-specific murine mAbs in terms of toxin-neutralizing activity. As only a few RTB-specific toxin-neutralizing antibodies have been identified, we deemed it important to characterize each of them as fully as possible as a means to identify which specific attributes (*e.g.*, affinity, epitope specificity, inhibition of attachment) are critical for antitoxin activity. Therefore, in this study we undertook a detailed *in vitro* characterization of RTB-B7.

## Materials and Methods

### 2.1 Chemicals, Biological Reagents and Cell Lines

Ricin toxin (*Ricinus communis* agglutinin II), FITC (Fluorescein isothiocyanate)-labeled ricin, biotinylated ricin toxin, *Ricinus communis* agglutinin I (RCA-I) and ricin toxin B subunit (RTB) were purchased from Vector Laboratories (Burlingame, CA). Ricin was dialyzed as described [29] against phosphate buffered saline (PBS) at 4°C in 10,000 MW cutoff Slide-A-Lyzer dialysis cassettes (Pierce, Rockford, IL), prior to use in cytotoxicity studies. D-(+)- Lactose, was obtained from J.T. Baker (Center Valley, PA) and asialofetuin (ASF) from Sigma-Aldrich (St. Louis, MO). Goat serum was purchased from Gibco-Invitrogen (Carlsbad, CA). Anti-E-tag Horseradish peroxidase (HRP) conjugated mAb was purchased from Bethyl Laboratories, Inc (Montgomery, TX) and goat-anti-mouse IgG HRP conjugated and streptavidin HRP conjugated were purchased from Fisher Scientific (Pittsburgh, PA). Unless noted otherwise, all other chemicals were obtained from Sigma-Aldrich (St. Louis, MO). Cell lines and cell culture media were obtained from the tissue culture media core facility at the Wadsworth Center. THP-1 cells were grown in RPMI +10% Fetal Bovine Serum (FBS) and Vero cells, a fibroblast-like kidney cell line derived from the African green monkey, were grown in DMEM +10% FBS. All cell lines were maintained in 37°C with 5% CO_2_ incubators, unless noted otherwise. Single chain camelid antibodies (V_H_Hs) which were E-tagged for ELISA purposes have been previously described [39] (**Table 1**).

**Table 1:** Characteristics of RTB-specific V_H_Hs and mAbs used in this study.

V_H_H/mAbs	GenBank^a^	Length (AA)	Vero*^b^*	THP-1 Pre*^c^*	THP-1 Post*^c^*	Protection
TFTB-1*^d^*	NA	NA	ND	ND	ND	–
24B11*^d^*	NA	NA	1.2	0.25	0.25	+
RTB-B7	KF746034	132	1.5	0.25	1.4	–
RTB-D12	KF746031	130	–	–	330	–
RTB-D8	KF746036	124	–	–	330	–
RTA-G12	KF746020	137	ND	ND	ND	–

a GenBank accession numbers; *^b^*Vero cytotoxicity assay performed as described in Materials and Methods; value shown are in nM; *^c^*Pre-bound and post-bound THP-1 cytotoxicity assays performed in Materials and Methods; values shown are in nM. *^d^*Anti-RTB mAbs previously characterized in [26], [29]. Abbreviations: ND, not determined; NA, not applicable.

### 2.2 Mouse Strains, Animal Care and Ricin Toxin Challenge Studies

Mouse experiments were performed as described [39]. Briefly, groups of female BALB/c mice (5 mice per group) approximately 8–10 weeks of age were purchased from Taconic Labs (Hudson, NY). Animals were housed under conventional, specific pathogen-free conditions and were treated in compliance with the Wadsworth Center’s Institutional Animal Care and Use Committee (IACUC) guidelines. For the challenge experiments, mice were injected by the intraperitoneal (i.p.) route on day 0 with pre-mixed ricin toxin (RT; 2 µg per mouse) and the corresponding V_H_H (RTB-B7 at 20 µg and 100 µg per mouse; RTB-D8 and RTB-D12 at 20 µg per mouse; D10/B7 at 30 µg per mouse) in a final volume of 0.4 mL PBS. Following challenge mice were monitored at least two times per day for overt signs of discomfort, including lethargy, hunching, and failure to resume normal feeding behavior, ruffled fur and absence of grooming. In addition, every 24 h, mice were assessed for the onset of hypoglycemia, a well-established surrogate marker of ricin intoxication. A drop of blood (5 µl) was collected from the lateral tail vein of each animal and blood glucose levels were measured using a hand-held glucometer (Accu-Chek Advantage, Roche, Indianapolis, IN). Mice were euthanized by carbon dioxide (CO_2_) asphyxiation when they became overtly moribund and/or blood glucose levels fell below 25 mg/dL. At no point in the study were the animals administered analgesics or anesthetics so as not to confound the effects of the antibody treatment. Statistical analysis was carried out using GraphPad Prism 5 (GraphPad Software, San Diego, CA). For survival studies, statistical significance was determined using the Log-Rank (Mantel-Cox) test.

### 2.3 Ethics Statement

Experiments described in this study that involved mice were reviewed and approved by the Wadsworth Center’s IACUC under protocol #13-384. The Wadsworth Center complies with the Public Health Service Policy on Humane Care and Use of Laboratory Animals and was issued assurance number A3183-01. Moreover, the Wadsworth Center is fully accredited by the Association for Assessment and Accreditation of Laboratory Animal Care (AAALAC). Obtaining this voluntary accreditation status reflects that Wadsworth Center’s Animal Care and Use Program meets all of the standards required by law, and goes beyond the standards as it strives to achieve excellence in animal care and use.

### 2.4 ELISAs for Determining V_H_Hs Specificity

ELISAs were performed as described [29]. Briefly, Nunc Immuno MicroWell 96 well plates from ThermoFisher Scientific (Rochester, NY) were coated overnight with 0.1 µg/well of ricin (15 nM), RTB (29 nM), RCA-I (16 nM) or 0.4 µg/well of ASF (82 nM) all in PBS (pH 7.4). The following day the plates were blocked with 2% PBS-goat serum (pH 7.4) for 2 h, then plates were treated with antibodies (V_H_Hs at 10 µg/mL, 330 nM; 24B11 at 22.75 µg/mL, 151.67 nM; TFTB-1 at 2 µg/mL, 13.33 nM) in 5-or 2-fold serial dilutions or with 0.1 µg/well of bovine serum albumin (BSA) (8 nM) for 1 h. For competition ELISAs, the antibodies were pre-mixed with ricin at 200 µg/mL and incubated for at least 30 min prior to adding to the plates for 1 h. Secondary antibodies, HRP-anti-E-tag, HRP-goat-anti-mouse IgG, or avidin-HRP were incubated for 1 h and developed using SureBlue Peroxidase Substrate, TMB (KPL, Gaithersburg, MD). The reactions were stopped using 1 M phosphoric acid and absorbance was read at 450 nm using the VersaMax Microplate Reader with Softmax Pro 5.2 software (Molecular Devices, Sunnyvale, CA). All samples were performed at least in triplicate.

### 2.5 Vero Cell Cytotoxicity Assays

Vero cell cytotoxicity assays were performed as described [29]. In brief, Vero cells were trypsinized, adjusted to ∼5 ×10^4^ cells per mL and seeded (100 µl/well) into white bottom 96-well plates (Corning Life Sciences, Corning, NY), and allowed to adhere overnight. Vero cells were then treated with ricin (0.01 µg/mL; 154 pM), ricin:Ab mixtures, or medium alone (negative control) for 2 h at 37°C. Cells were washed to remove non-internalized toxin or ricin:Ab mixtures, and 100 µl of fresh medium was added to the wells. Fresh medium was allowed to incubate for 48 h and cell viability was measured using CellTiter-Glo (Promega, Madison, WI). All samples were performed in quadruplicate and 100% viability was defined as the average value obtained from wells in which cells were treated with medium only.

### 2.6 THP-1 Cell Cytotoxicity Assays

THP-1 cell cytotoxicity assays were done as described [40]. Briefly, THP-1 cells were spun (5 min at 400×*g*) and adjusted to ∼5 ×10^4^ cells per mL and seeded (100 µl/well) into clear U-bottom 96-well plates (BD Bioscience, San Jose CA) and allowed to grow overnight. The next day, THP-1 cells were spun to remove medium and were then treated with ricin (0.01 µg/mL; 154 pM), ricin:Ab mixtures, or medium alone for 2 h at 37°C. Cells were then subjected to centrifugation and washed to remove non-internalized toxin or ricin:Ab mixtures. Fresh medium was added to the wells and allowed to incubate for 48 h. Cell viability was determined using CellTiter-Glo after the content of each plate was transferred into white bottom 96-well plates. In order to address post-attachment experiments, THP-1 cells were kept on ice at 4°C, to allow for ricin attachment but prevented internalization of the toxin prior to antibody treatment. The cytotoxicity assay was performed as described above but cells were kept on ice until they were transferred to 37°C for the 48 h incubation period. All samples were performed in quadruplicate and 100% viability was defined as the average value obtained from wells in which cells were treated with medium only.

### 2.7 Ricin Binding Assays using THP-1 Cells and Flow Cytometry

Ricin binding to cell surfaces was performed as described [26]. In brief, THP-1 cells were collected by gentle, low speed centrifugation (5 min at 400×*g*). The resulting cell pellets were suspended to ∼5 ×10^6^ cells per mL and then seeded (200 µl/well) into clear U-bottom 96-well plates (BD Bioscience, San Jose CA). FITC-labeled ricin (3 µg/mL) was mixed with Abs or lactose (30 mg/mL) for 30 min on ice in the dark prior to being added to THP-1 cells. Cells were then washed twice with PHEM buffer to remove unbound toxin:Ab complexes and fixed with 4% paraformaldehyde (PFA) in PHEM for 15 min. Ricin binding to the surfaces of THP-1 cells was measured using FACS Calibur flow cytometer (BD Bioscience, San Jose CA). A minimum of 10,000 events was analyzed per sample.

### 2.8 Analytical Ultracentrifugation (AUC)

Ricin and Ab samples were dialyzed overnight in PBS (pH 7.4) at 4°C in 10,000 MW cutoff Slide-A-Lyzer dialysis cassettes (Pierce, Rockford, IL) prior to being subject to AUC. Sedimentation Velocity (SV) experiments were conducted in a Beckman Optima XL-I analytical ultracentrifuge at 20°C at a rotor speed of 50,000 rpm. Double-sector charcoal-filled epon centerpieces were filled with a sample volume of 400 µl and the reference volume of dialysis buffer was 420 µl. Absorption measurements were made at 280 nm or 230 nm, dependending on the protein concentration. Samples were run in an An-60 Ti four-hole rotor with zero time between scans. Care was taken to have the rotor at thermal equilibrium for an hour before accelerating directly to the speed of the experiment. The data were analyzed by the c(s) method found in SEDFIT [41]. The experimentally calculated sedimentation coefficients were converted to s20,w values within the SEDFIT software and graphed using Origins (OriginLab Corporation, Northampton, MA).

### 2.9 Statistical Analyses and Modeling Software

Statistical analysis was carried out using GraphPad Prism 5 (GraphPad Software, San Diego, CA). The open-source molecular visualization software PyMOL (DeLano Scientific LLC, Palo Alto, CA) was used for modeling of ricin.

## Results

### 3.1 Passive Protection Studies with V_H_H RTB-B7

We previously reported that three different heterodimeric V_H_Hs, each containing RTB-B7, were capable of passively protecting mice against ricin toxin [39]. However, in that same study we reported that monomeric RTB-B7 (10 µg per mouse; 10∶1 V_H_H:toxin molar ratio) afforded no benefit to mice in the face of a 10xLD_50_ ricin challenge. To investigate whether the failure of RTB-B7 to passively protect mice was simply an issue of insufficient antibody, we repeated the passive protection studies using 2-fold (20 µg) and 10-fold (100 µg) higher amounts of RTB-B7. For comparison purposes, two other RTB-specific V_H_Hs were tested in parallel: RTB-D8 and RTB-D12. We chose RTB-D12 because its apparent affinity (EC_50_) for ricin toxin, as determined by ELISA, is virtually identical to that of RTB-B7’s (0.8 nM versus 0.6 nM, respectively). RTB-D8 was chosen because it binds to ricin with a relatively low affinity (EC_50_ 3.6 nM) and was therefore not expected to provide an *in vivo* toxin-neutralizing activity. As a positive control for these studies, groups of mice were treated with the V_H_H heterodimer D10/B7. Following ricin challenge, animals were monitored for a period of 7 days for the onset of hypoglycemia, a well-established surrogate marker of ricin intoxication, as well as mortality.

**Figure 2:**
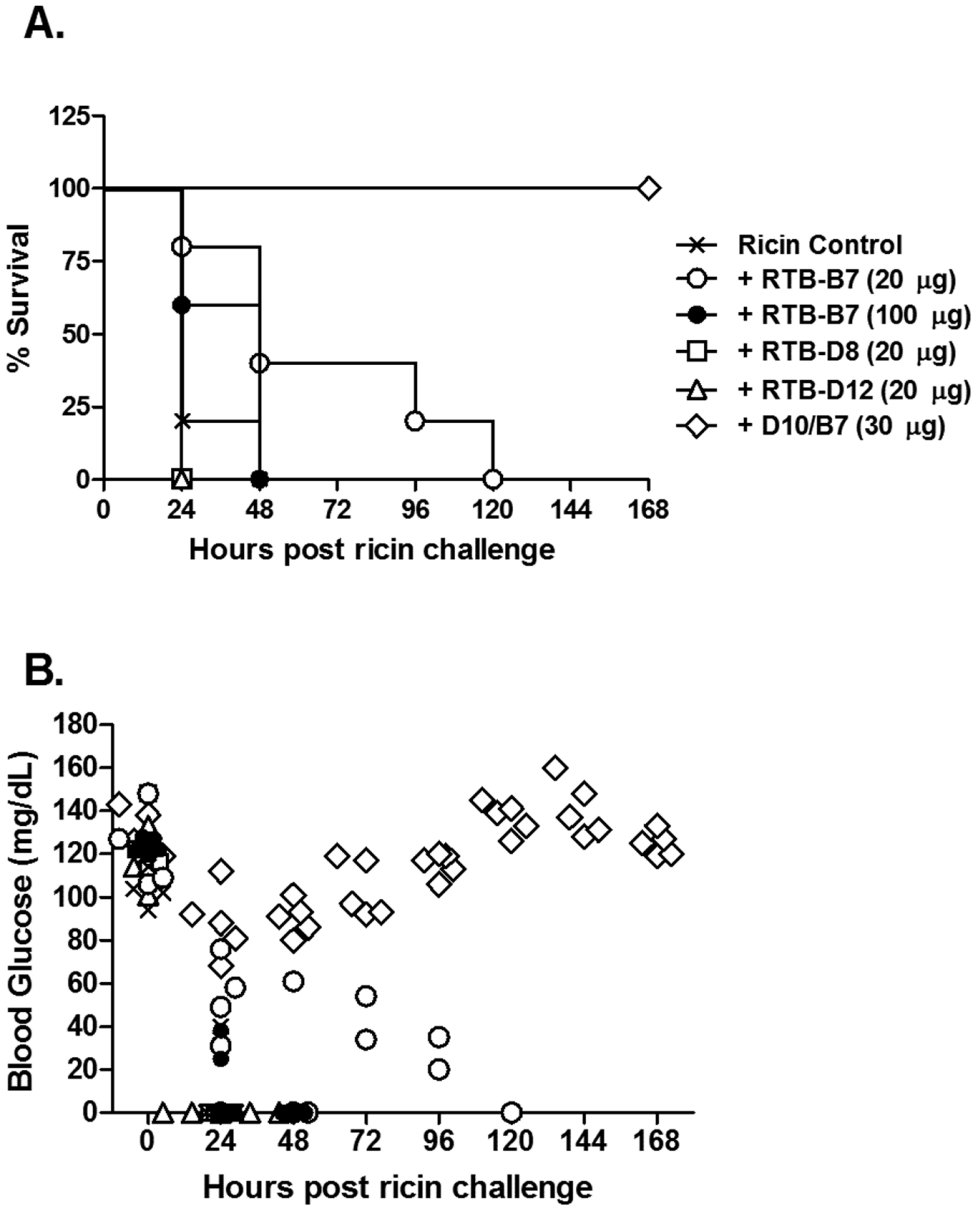
Monomeric RTB-B7 does not passively protective mice from ricin challenge. Antibodies D10/B7, RTB-B7, RTB-D8 or RTB-D12 were mixed with ricin (2 µg; equivalent to 10xLD_50_) and then administered to adult BALB/c mice (n = 5 per group) by i.p. injection. Mice were monitored for seven days for (A) survival and (B) hypoglycemia.

As expected, mice that received the heterodimer D10/B7 (30 µg; 20∶1 V_H_H heterodimer:toxin molar ratio) survived ricin challenge and experienced only minor declines in blood glucose levels (**Fig. 2**). Conversely, neither RTB-D8 nor RTB-D12 (each at 20 µg; 20∶1 V_H_H:toxin molar ratio) afforded any protection against ricin toxin, as evidenced by the fact that the V_H_H-treated mice experienced a rapid decline in blood glucose levels and died within 24 h (**Fig. 2**). Surprisingly, even relatively high amounts of RTB-B7 (20 µg and 100 µg, equivalent to 20∶1 and 100∶1 V_H_H:toxin molar ratio, respectively) had no impact on survival (0/5 mice survived in both groups) following ricin challenge (**Fig. 2**). Overall, these data demonstrate that RTB-B7, as a monomer, does not elicit protection *in vivo*, but as a heterodimer (D10/B7) is capable of passively protecting mice against 10xLD_50_ of ricin.

### 3.2 RTB-B7 Neutralizes Ricin in vitro as Effectively as 24B11

**Figure 3:**
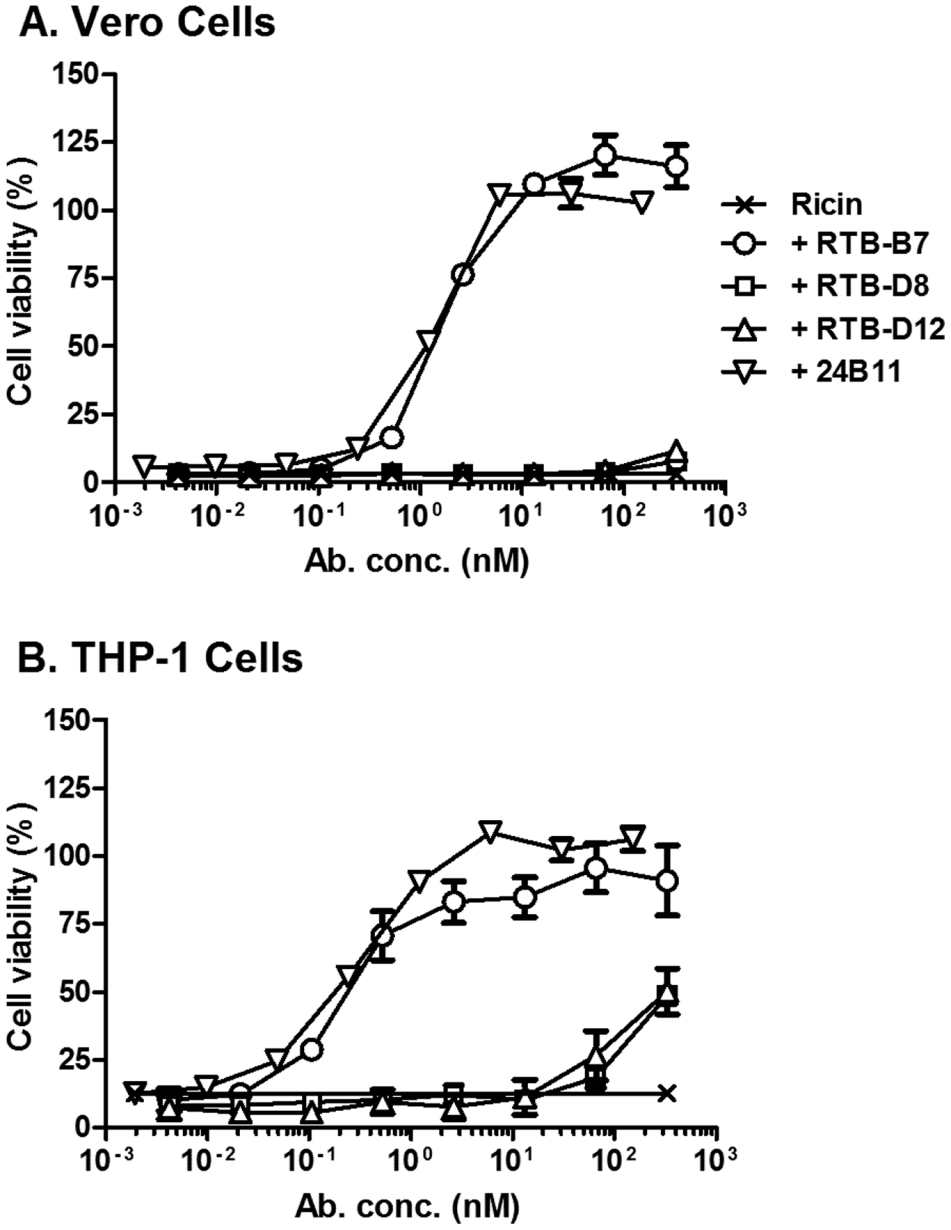
RTB-B7 neutralizes ricin in a dose-dependent manner. Ricin was mixed with 2-fold serial dilutions of indicated antibodies and then applied to (A) Vero cells or (B) THP-1 cells for 2 h. The cells were then washed and cell viability was measured 48 h later, as described in Materials and Methods. The data (mean ± SD) represent a single experiment in which each sample was done in quadruplicate. The experiment was repeated at least twice with identical results.

The inability of RTB-B7 (even at 100 fold molar excess over ricin) to passively protect mice against a 10xLD_50_ ricin challenge was unexpected, considering that we previously reported that RTB-B7 was highly effective at neutralizing ricin in a Vero cell cytotoxicity assay [39]. We therefore revisited the Vero cell cytotoxicity assay and compared RTB-B7 side-by-side with 24B11, as well as the non-neutralizing V_H_Hs, RTB-D8 and RTB-D12. As reported previously, we found that RTB-B7 neutralized ricin in a dose-dependent manner. Moreover, RTB-B7’s estimated IC_50_ (∼1.5 nM) was virtually identical to 24B11’s IC_50_ when the two antibodies were compared in the same assay (**Fig. 3A**). In contrast, neither RTB-D8 nor RTB-D12 (which has the same apparent affinity for ricin as RTB-B7) had any detectable toxin-neutralizing activity (**Fig. 3A**).

We next examined the capacity of RTB-B7 to neutralize ricin *in vitro* using THP-1 cells, a human monocyte/macrophage-derived cell line that is potentially more representative of ricin’s primary target cell *in vivo* [42], [43]. In the THP-1 assay, RTB-B7 also neutralized ricin with a dose-dependent profile that was nearly identical to 24B11’s (**Fig. 3B**), thereby demonstrating that RTB-B7 is among the most potent *in vitro* toxin-neutralizing antibodies described to date. In the THP-1 assay, RTB-D8 and RTB-D12 had some detectable toxin-neutralizing activity at very high concentrations, suggesting these two V_H_Hs should be classified as partially neutralizing (not non-neutralizing) antibodies (**Fig. 3B**).

### 3.3 Characteristics of RTB-B7 Recognition of Ricin and RTB

The capacity of RTB-B7 to neutralize ricin *in vitro* but not *in vivo* led us to investigate in more detail the specific *in vitro* properties of RTB-B7, particularly with respect to specificity of toxin binding and recognition. In an effort to define the epitope recognized by RTB-B7, we subjected RTB-B7 (as well as RTB-D8 and RTB-D12) to the following previously established assays: RCA-I ELISA, pepscan analysis [29], panning with a 12-mer phage-displayed peptide library [28], [44], Western blot analysis, and competitive ELISAs with a collection of well-characterized RTB-specific neutralizing and non-neutralizing murine mAbs [26].

RCA-I is a tetrameric glycoprotein from *Ricinus communis* consisting of two ricin-like heterodimers whose B subunit (RCB) shares 84% sequence identity with RTB [29], [45], [46]. We found that RTB-B7 bound ricin and RCA-I with similar EC_50_s, demonstrating that RTB-B7’s epitope is likely conserved between the two closely related proteins (**Fig. S1**). RTB-D8 and RTB-D12 were similar to RTB-B7 in that they bound equally well to RCA-I and ricin. However, RTB-B7’s epitope is likely discontinuous in nature, as neither pepscan or affinity enrichment using a 12-mer phage-displayed library identified peptides that specifically bound RTB-B7. Moreover, Western blot analysis indicated that RTB-B7 reactivity was abolished when ricin (or RTB) was treated with β-mercaptoethanol (BME) (**data not shown**).

It was previously reported that neither SylH3 nor 24B11, two RTB-specific neutralizing mAbs, were able to competitively inhibit RTB-B7 from binding to ricin [39]. We extended these previous findings by performing competitive ELISAs with additional neutralizing (JB4) and non-neutralizing (TFTB-1, B/J F9, C/M A2, SA3, CB12, and JB11) murine mAbs. Ricin-coated ELISA plates were incubated with saturating amounts of murine mAbs and then probed with the RTB-B7, RTB-D8 or RTB-D12 (1 µg/mL; 33 nM). The EC_50_s of RTB-B7, RTB-D8 and RTB-D12 were unaffected by any of the murine mAbs tested (**data not shown**), suggesting that the three V_H_Hs recognize distinct epitopes on RTB.

**Figure 4:**
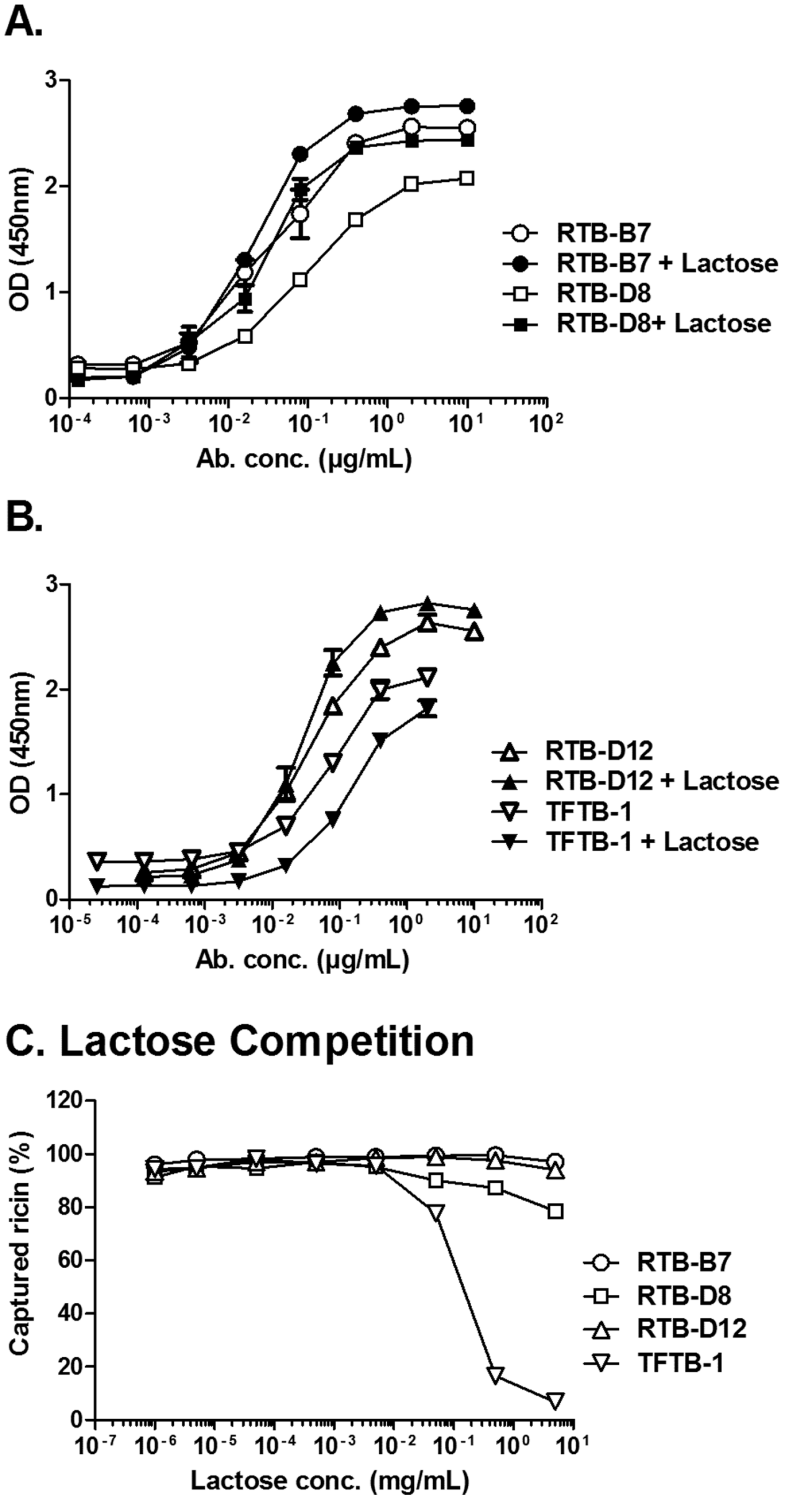
Impact of lactose on RTB-B7’s recognition of ricin. To examine the impact of lactose on the ability of antibody to recognize ricin, ELISA plates were coated with ricin overnight. Plates were then probed with lactose for 1-mixed lactose with serial diluted concentrations of (A) RTB-B7 and RTB-D8 or (B) RTB-D12 and TFTB-1. Plates were incubated with mixtures of lactose:Ab and developed as described in Materials and Methods. (C) In the competition ELISA, plates were coated with the indicated antibodies at a constant concentration (V_H_Hs at 10 µg/mL and TFTB-1 at 2 µg/mL) overnight. Serial dilutions of lactose (5 mg/mL) were incubated with biotinylated ricin before being applied to plates and developed. Binding was normalized to ricin bound to antibody in the absence of lactose. The data shown represent a single experiment in which each sample was done in triplicate and repeated at least twice. Data are expressed as the mean ± SD.

To determine whether RTB-B7 recognizes an epitope on RTB that is influenced by ligand engagement, we performed ricin ELISAs in the absence or presence of saturating amounts of lactose (10 mg/mL). The apparent affinity of RTB-B7 for ricin was unchanged (or even slightly enhanced) in the presence of lactose (**Fig. 4A, B**). This is in contrast to the non-neutralizing murine mAb, TFTB-1, whose capacity to recognize ricin was negatively impacted (∼20%) by lactose (**Fig. 4B**). The effect of ligand binding was even more pronounced when the antibodies were tested for their ability to “capture” soluble, biotinylated ricin in the presence of increasing concentrations of lactose (**Fig. 4C**). Lactose concentrations greater than 0.1 mg/mL resulted in a precipitous drop in TFTB-1′s ability to capture soluble ricin, whereas the abilities of V_H_Hs RTB-B7, RTB-D8 and RTB-D12 to capture ricin were not altered (**Fig. 4C**). These data indicate that RTB-B7 recognizes an epitope on RTB that is not influenced by ligand engagement.

**Figure 5:**
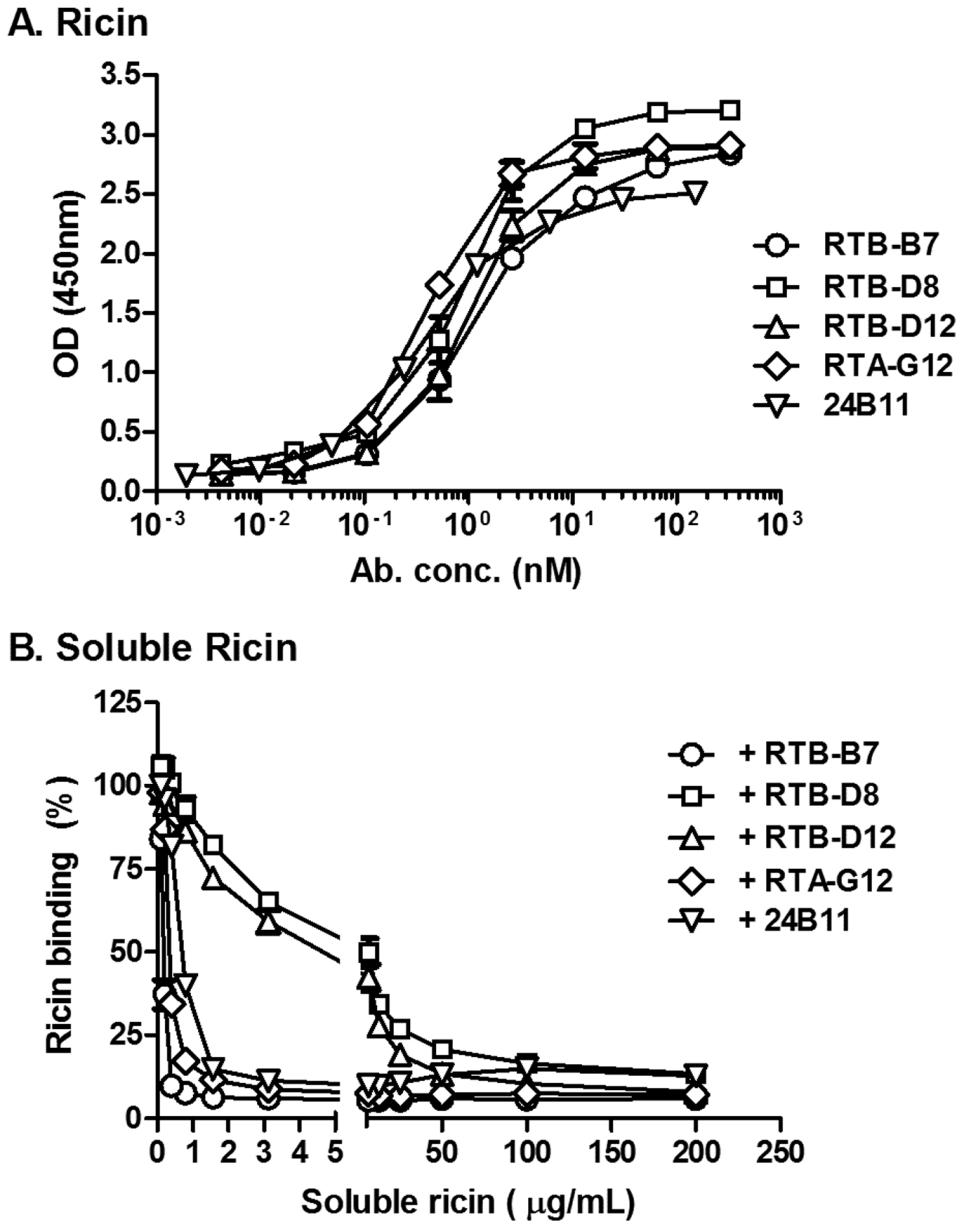
Recognition of plate bound and soluble ricin by RTB-B7. (A) ELISA plates were coated overnight with ricin. Antibodies 24B11 and V_H_Hs were serial diluted and added to the coated plates and developed as described in Materials and Methods. Shown on the Y-axis is optical density (OD). (B) For the competition ELISA, plates were coated with ricin overnight. Ricin (200 µg/mL) was serial diluted and pre-mixed with constant concentration of the indicated antibodies at equal molar amounts taking into account the number of binding sites (V_H_Hs at 10 µg/mL and 24B11 at 22.75 µg/mL). Ricin:Ab solutions were then added to the plates and developed. The data shown represent a single experiment in which each sample was done in triplicate and repeated at least twice. Data are expressed as the mean ± SD.

We have recently noted that certain mAbs recognize plate-bound ricin with high affinity, but bind poorly to ricin in solution (J. O’Hara, A. Yermakova, E. Sully, and N. Mantis, *manuscript in preparation*). Based on these and other observations, we speculate that adsorption of ricin to polystyrene microtiter plates results in the exposure of normally cryptic or subdominant epitopes (R’-form). To examine accessibility of RTB-B7’s epitope, we performed ELISAs in the absence or presence of soluble ricin toxin (**Figure 5**). Antibodies 24B11 and RTA-G12, an RTA-specific V_H_H with toxin-neutralizing activity, were examined in parallel. We confirmed that RTB-B7, RTB-D8 and RTB-D12, as well as 24B11 and RTA-G12, recognized plate-bound ricin with roughly equal EC_50_s (**Figure 5A**). On the other hand, attachment of RTB-B7, 24B11 and RTA-G12 to plate bound ricin was completely inhibited by very low amounts of soluble native ricin (IC_50_<0.1 µg/mL), whereas inhibition of RTB-D8 and RTB-D12 required very high concentrations (>50 µg/ml) of toxin (**Figure 5B**). These data demonstrate that RTB-B7’s epitope is presented on the surface of native ricin, whereas the epitopes recognized by RTB-D8 and RTB-D12 are significantly (>500 fold) less accessible.

### 3.4 RTB-B7 is Able to Neutralize Ricin after the Toxin has attached to Host Cell Receptors

**Figure 6:**
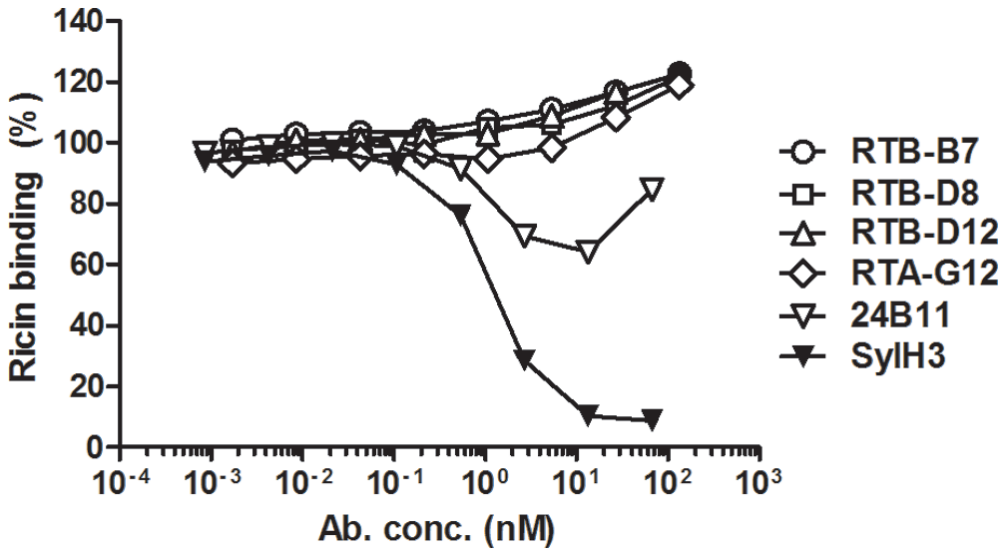
RTB-B7 does not inhibit ricin from biding to ASF. ELISA plates where coated with ASF overnight. Plates were then probed with pre-mixed ricin with the indicated V_H_Hs (4.4 µg/mL) or mAbs 24B11 and SylH3 (10 µg/mL). Binding was normalized to ricin in the absence of antibody. The data shown represent a single experiment in which each sample was done in triplicate and repeated at least twice. Data are expressed as the mean ± SD. In most instances error bars are masked by symbol and therefore not visible.

There is evidence that RTB-specific toxin-neutralizing antibodies can be loosely classified into one of two categories based on the degree to which they prevent ricin from binding to cellular receptors and whether or not they neutralize ricin after the toxin has pre-bound to host cells [21], [23], [25]–[29]. For example, SylH3 is a potent inhibitor of ricin-receptor interactions but only marginally effective at neutralizing ricin when pre-bound to cell surfaces, while 24B11 only partially inhibits toxin-receptor interactions but is highly effective at neutralizing ricin when toxin is pre-bound to cells [27], [28]. In an effort to better characterize RTB-B7, we first examined its ability to inhibit ricin from binding to the surrogate receptor ASF. Biotinylated ricin was mixed with RTB-B7 at a range of concentrations and then applied to ELISA plates coated with ASF. SylH3 and 24B11, as well as RTB-D8 and RTB-D12, were examined in parallel. As expected, SylH3 almost completely blocked (>90%; EC_50_ ∼1 nM) the ability of ricin to bind to ASF, while 24B11 only marginally (<30%) impacted toxin-ASF interactions (**Fig. 6**). In contrast, neither RTB-B7 nor the partially neutralizing V_H_Hs, RTB-D8 and RTB-D12, affected the ability of ricin to associate with ASF, even at the highest concentrations tested.

To assess antibody effects on ricin’s interactions with cell surfaces, FITC-ricin was incubated with approximately equimolar amounts of RTB-B7 or other V_H_Hs/mAbs and then applied to THP-1 cells at 4°C. The cells were then washed and subjected to flow cytometry to quantitate the amount of FITC-ricin bound to the cells. Lactose (30 mg/mL; 88 mM) was used as a positive control for these studies, as saturating amounts of the disaccharide are known to occupy RTB’s carbohydrate recognition domains and competitively inhibit ricin binding to cell surfaces [7]. The non-neutralizing mAb TFTB-1 was used as a negative control [26]. As expected, lactose inhibited ricin from binding to THP-1 cells, whereas TFTB-1 did not (<5%) (**Table 2**). V_H_Hs RTB-D8, RTB-D12 and RTB-B7 partially (20–30%) inhibited ricin from binding to THP-1 cells, while D10/B7 and 24B11 reduced ricin binding to THP-1 cells by >85% and >65%, respectively. Thus, RTB-B7 does not inhibit ricin binding to cell surfaces as efficiently as D10/B7.

**Table 2:** Antibody-mediated inhibition of ricin binding to the surface of THP-1 cells.

Ricin	Treatment	Conc.	Inhibition (%)^a^
+	–	–	0
+	Lactose	88 mM	75
+	RTB-B7	50 nM	29
+	RTB-D8	50 nM	18
+	RTB-D12	50 nM	28
+	D10/B7	50 nM	86
+	24B11	50 nM	66
+	TFTB-1	50 nM	2.5

a FITC-ricin (46 nM) was incubated with lactose or indicated antibodies for 30 min before being applied to THP-1 cells and then subjected to flow cytometry, as described in Materials and Methods. The percent (%) inhibition of ricin binding was calculated by dividing the experimental geometric mean fluorescence intensity (MFI) by the control (FITC-ricin only) geometric MFI and then multiplying by 100.

**Figure 7:**
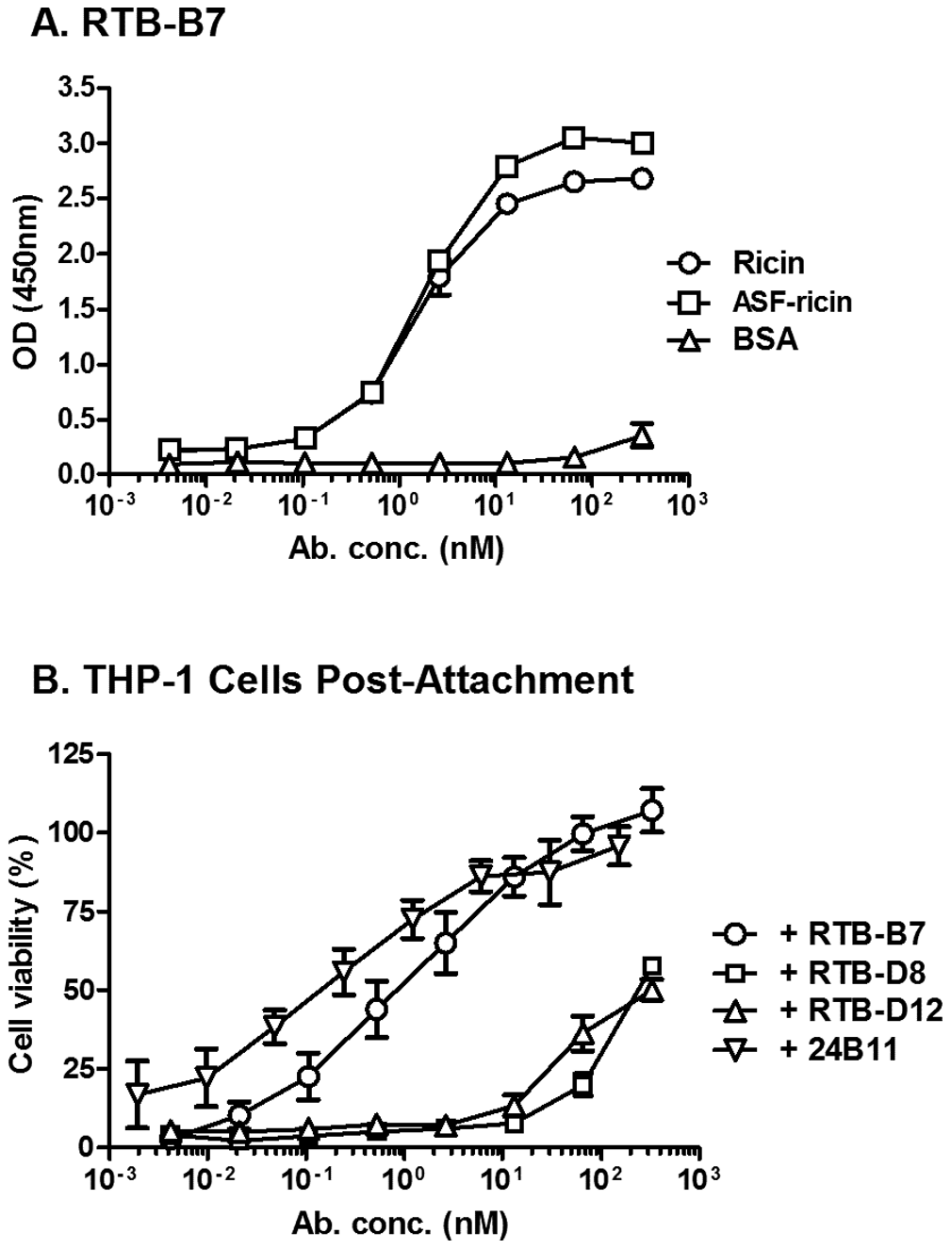
RTB-B7 neutralizes ricin when toxin is pre-bound to receptor. (A) To examine antibody recognition of receptor bound ricin, ELISA plates were coated with ricin or ASF overnight. ASF coated plates where then incubated with ricin while the remaining wells were incubated with 2% PBS-goat serum. RTB-B7 was serial diluted and added to the wells and developed as described in Materials and Methods. BSA wells were used to control for background. (B) To examine toxin neutralizing activity, THP-1 cells were treated with ricin for 30 min at 4°C, then washed and incubated with antibody or medium alone for 30 min at 4°C. Then, THP-1 cells where transferred to 37°C and 48 h later cell viability was measured as described in Materials and Methods. Viability was normalized to medium only treated cells and ricin-only treated cells determined the potency of the toxin in this assay. The ricin-only control cells were <20% viable. The data shown represent a single experiment in which each sample was done in triplicate or quadruplicate and repeated at least twice. Data are expressed as the mean ± SD.

We next examined the ability of RTB-B7 to recognize receptor-bound ricin and to neutralize toxin when pre-bound to cell surfaces. ELISA plates coated with ASF were incubated with a saturating concentration of ricin and then probed with RTB-B7 across a range of concentrations. RTB-B7 (**Fig. 7A**), as well as RTB-D8, RTB-D12 and D10/B7 (**data not shown**), recognized ASF-ricin in a dose-dependent manner. RTB-B7 was next tested for the ability to neutralize ricin after the toxin was pre-bound to cell surfaces. In order to do so, THP-1 cells were cooled on ice to arrest toxin endocytosis and treated with ricin for 30 min before the addition of RTB-B7, RTB-D8, RTB-D12 or 24B11, as described in the Materials and Methods. We found that 24B11 (IC_50_ 0.25 nM) and RTB-B7 (IC_50_ 1.4 nM) were each able to effectively neutralize ricin in this assay (**Fig. 7B**), whereas RTB-D8 and RTB-D12 were relatively ineffective, as they had detectable toxin-neutralizing activities only at very high concentrations (>330 nM) (**Fig. 7B**). These data demonstrate that RTB-B7, like 24B11, has the capacity to neutralize ricin after the toxin has adhered to cell surface receptors.

### 3.6 Antibodies D10/B7 and 24B11 Promote Ricin Aggregation in Solution, Whereas RTB-B7 does not

**Figure 8:**
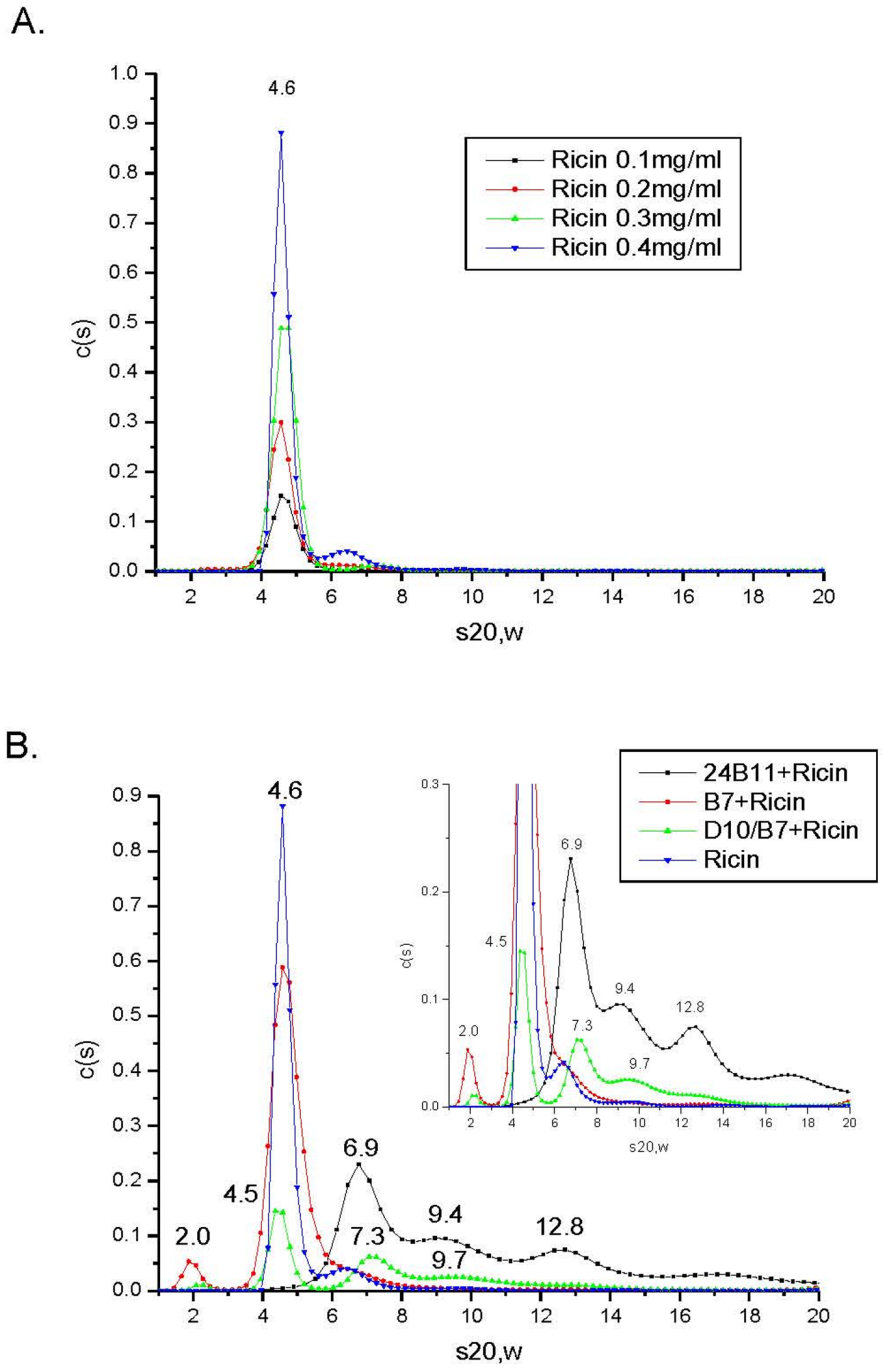
Sedimentation coefficients for ricin and ricin:antibody complexes. Ricin and ricin:Ab complexes were subject to AUC, as described in the Materials and Methods. (A) Sedimentation coefficients of ricin were determined at indicated toxin concentrations (0.1–0.4 mg/ml). (B) Sedimentation coefficients of ricin:Ab complexes. Ricin was mixed with 24B11 (3.5 µM), D10/B7 (3.5 µM) and RTB-B7 (7 µM) to achieve an equimolar ratio between ricin and ricin-binding sites on each of the three different antibodies. The mixtures were incubated at room temperature for 120–180 min and then subjected to AUC (20°C). For convenience, the corrected sedimentation coefficients (s20,w) are denoted above each sedimentation distribution in the plot. The inset in panel B is simply a magnification of the figure sedimentation profiles to enhance resolution of the X-axis.

V_H_H heterodimer D10/B7 consists of two ricin-specific single chain V_H_H monomers, RTA-D10 and RTB-B7, joined by a flexible peptide linker [39]. By virtue of its ability to bind epitopes on RTA and RTB, D10/B7 can theoretically crosslink ricin and promote the formation of heterogeneous toxin-antibody complexes. Although monomeric in nature, we cannot formally exclude the possibility that RTB-B7 may homodimerize and therefore promote ricin aggregation to some degree. Because the formation of toxin:antibody complexes may contribute to toxin-neutralizing activity, we subjected our antibody samples to AUC analysis. Sedimentation coefficients for ricin, 24B11, D10/B7 and RTB-B7 were examined at a range of concentrations and were determined to be 4.6S, 6.7S, 2.9S and 2.3S, respectively **(** **Fig. 8 A** **;** **S2A–C**). The sedimentation coefficient for ricin (s20,w = 4.6S) was identical to what is reported in the literature [47].

In order to characterize the aggregation potential of the individual antibodies, RTB-B7, D10/B7 and 24B11 were mixed at a 1∶1 molar ratio with ricin and then subjected to AUC. As shown in **Figure 8B**, D10/B7 and 24B11 promoted ricin aggregation, whereas RTB-B7 did not. Specifically, ricin:24B11 mixtures resulted in three defined sedimentation coefficient distributions at 6.9S, 9.4S and 12.8S, which we interpreted as corresponding to 24B11 (s20,w = 6.9S), a 1∶1 ricin:24B11 complex (s20,w = 9.4S) and 2∶1 ricin:24B11 complex (s20,w = 12.8S). The sedimentation coefficients for ricin:D10/B7 mixture revealed distributions at 4.5S and 7.3S, followed by a long tail with a larger complex identified at 9.7S. We interpret this distribution profile as corresponding to free ricin (s20,w = 4.5S), a 1∶1 mixture of ricin:D10/B7 (s20, w = 7.3S), and heterogeneous ricin:D10/B7 aggregates of various sizes. Due to RTB-B7’s small size, its effect on ricin aggregation was difficult to discern. However, the peak distribution around 4.6S had a much broader shoulder than the ricin control sample, suggesting the presence of free ricin plus a 1∶1 ricin:RTB-B7 complexes, not higher in molecular weight complexes.

## Discussion

The antibody response to ricin toxin is surprisingly complex and the specific correlates of protection to this biothreat agent remain poorly defined, especially as compared to other protein toxins. For example, tetanus and diphtheria vaccines have been in clinical use for decades and it is well established that the primary correlate of protection is associated with a specific threshold of serum IgG toxin-neutralization activity [48]. In the case of ricin, however, protection (in mice) does not necessarily correlate with either pre-existing toxin-neutralizing activities or total antitoxin antibody levels [49], [50]. Similarly, analysis of serum IgG responses in humans that received a candidate ricin toxin subunit vaccine revealed a disconnect between total antibody titers and toxin-neutralizing activities (*i.e.*, several individuals with high ricin-specific serum IgG titers had low or undetectable toxin-neutralizing activities) [51], [52]. The absence of definitive correlates of immunity to ricin has hindered evaluation of candidate ricin subunit vaccines and made the development of immunotherapeutics challenging. Therefore, as a strategy to “deconvolve” the polyclonal antibody response to ricin, we have focused on characterizing a large number of murine mAbs and camelid nanobodies with the goal of identifying the specific determinants that are associated with toxin-neutralizing activity *in vivo* [39], [53].

In this study, we undertook a comprehensive *in vitro* characterization of the camelid antibody RTB-B7. RTB-B7 is of interest as it was the only antibody with toxin-neutralizing activity among a collection of nine recently described RTB-specific V_H_Hs [39]. Moreover, heterodimers consisting of RTB-B7 linked to one of three different RTA-specific V_H_Hs (*i.e*., RTA-D10) were able to passively protect mice against ricin challenge, even though 100-fold molar excess of monomeric RTB-B7 does not alter the toxicity of ricin *in vivo* (**Figure 2**). The main findings of the current study are that RTB-B7 (i) has potent *in vitro* toxin-neutralizing activity in two well-established cell-based cytotoxicity assays; (ii) recognizes a conformational epitope that is conserved between RTB and RCB (see below); (iii) binds to and neutralizes ricin in its soluble and receptor-bound forms; but, (iv) only partially inhibits ricin attachment to cell surface receptors. Thus, at least *in vitro*, RTB-B7 and 24B11 are virtually indistinguishable. Yet, 24B11 is able to passively protect mice against ricin toxin, whereas RTB-B7 is not [28]. The reason why RTB-B7 is devoid of detectable toxin-neutralizing activity *in vivo* remains unknown, but the fact that 24B11 Fab fragments are able to passively immunize mice demonstrates that neither antibody avidity or Fc-mediated clearance is absolutely required for protection *in vivo* [28]. Based on these data, we postulate that epitope specificity, and not affinity or avidity per se is the primary determinant of ricin toxin-neutralizing activity. The importance of epitope specificity in ricin toxin neutralization *in vivo* is supported as well by other studies with murine mAbs and V_H_H heterodimers [26], [39], [54].

While RTB-B7’s epitope was not definitively identified in this study, it can be tentatively localized to a limited region on RTB. For example, RTB-B7 bound equally well to RTB and RCB, indicating that RTB-B7’s epitope is conserved between the two proteins, which are only 84% identical at the amino acid level [45]. The differences between RTB and RCB are concentrated within domain 1, particularly within subdomains 1α and 1β, suggesting that RTB-B7 may recognize an epitope in RTB’s domain 2. Antibody competition studies with a collection of neutralizing (24B11, SylH3 and JB4) and non-neutralizing mAbs (TFTB-1, B/J F9, C/M A2, SA3, CB12 and JB11) is also consistent with RTB-B7 being targeted to domain 2, particularly a stretch of residues (200–240) within subdomain 2γ that is conserved between RTB and RCB. The fact that RTB-B7 was not particularly effective at inhibiting ricin attachment to cell surfaces would suggest that RTB-B7’s epitope is spatially distinct from key residues of RTB involved in Gal/GalNAc recognition (e.g., residues 248 and 234 in domain 2). Finally, in taking in account solvent accessibility, we postulate that RTB-B7 recognizes a conformational epitope localized within residues 200–230 of RTB.

It is interesting to note that the heterodimer V_H_H D10/B7, which consists of RTB-B7 covalently linked via a short peptide spacer to RTA-D10, was highly effective (>85%) at blocking attachment of ricin to the surface of THP-1 cells and, as AUC analysis revealed, promoting ricin aggregation in solution. It is tempting to speculate that one or both of these attributes are important in neutralizing ricin *in vivo*. RTA-D10 has moderate toxin-neutralizing activity *in vitro*, but like RTB-B7 is unable to protect mice against ricin challenge. We recently solved the X-ray crystal structure of RTA-D10 in complex with RTA (MJ Rudolph, DJ Vance, J Cheung, MC Franklin, F Burshteyn, MS Cassidy, EN Gary, C Herrera, CB Shoemaker, and NJ Mantis, *manuscript resubmitted*), revealing that RTA-D10 makes contact with a face of RTA that is almost diametrically opposed to RTB. Considering that the spacer between RTB-B7 and RTA-D10 is (at least) theoretically too short to enable intra-molecular RTA-RTB interaction, it is likely that D10/B7 promotes the formation of ricin intermolecular crosslinking, which is consistent with the aggregation seen in the AUC experiments. It is unclear at this point whether toxin aggregation and inhibition of toxin binding to cell surfaces are separate phenomena or whether aggregation itself results in the inability of ricin to attach to membrane bound Gal/GalNAc moieties. In future studies we will systematically construct and characterize additional anti-ricin V_H_H heterodimers and compare them with their respective monomeric constitutes as a means to dissect the functional properties of antibodies that are important in toxin neutralization *in vivo*. Only then will it be possible to rationally design effective therapeutics against ricin.

## Supporting Information

Figure S1 **Reactivity of RTB-B7, RTB-D8 and RTB-D12 with RCA-I.** ELISA plates were coated with ricin or RCA-I overnight. (A) RTB-B7, (B) RTB-D8 and (C) RTB-D12 were serial diluted and added to the wells and reactivity was determined as mentioned in Materials and Methods. BSA wells were used to control for background. The data shown represent a single experiment in which each sample was done in triplicate and repeated at least twice. Data are expressed as the mean ± SD.
(TIF)

Figure S2 **Sedimentation coefficients for 24B11, D10/B7 and RTB-B7.** AUC was used to determine sedimentation coefficients for antibodies (A) RTB-B7, (B) D10/B7 and (B) 24B11 at indicated concentrations, as described in the Materials and Methods. For convenience, the corrected sedimentation coefficients (s20,w) are denoted above each sedimentation distribution.
(TIFF)
