# Supplementary figures and images for: Differential Neutralizing Activities of a Single Domain Camelid Antibody (VHH) Specific for Ricin Toxin’s Binding Subunit (RTB)
Source: PLoS One. 2014 Jun 11;9(6):e99788. doi: 10.1371/journal.pone.0099788 (PMC4053406; doi:10.1371/journal.pone.0099788)

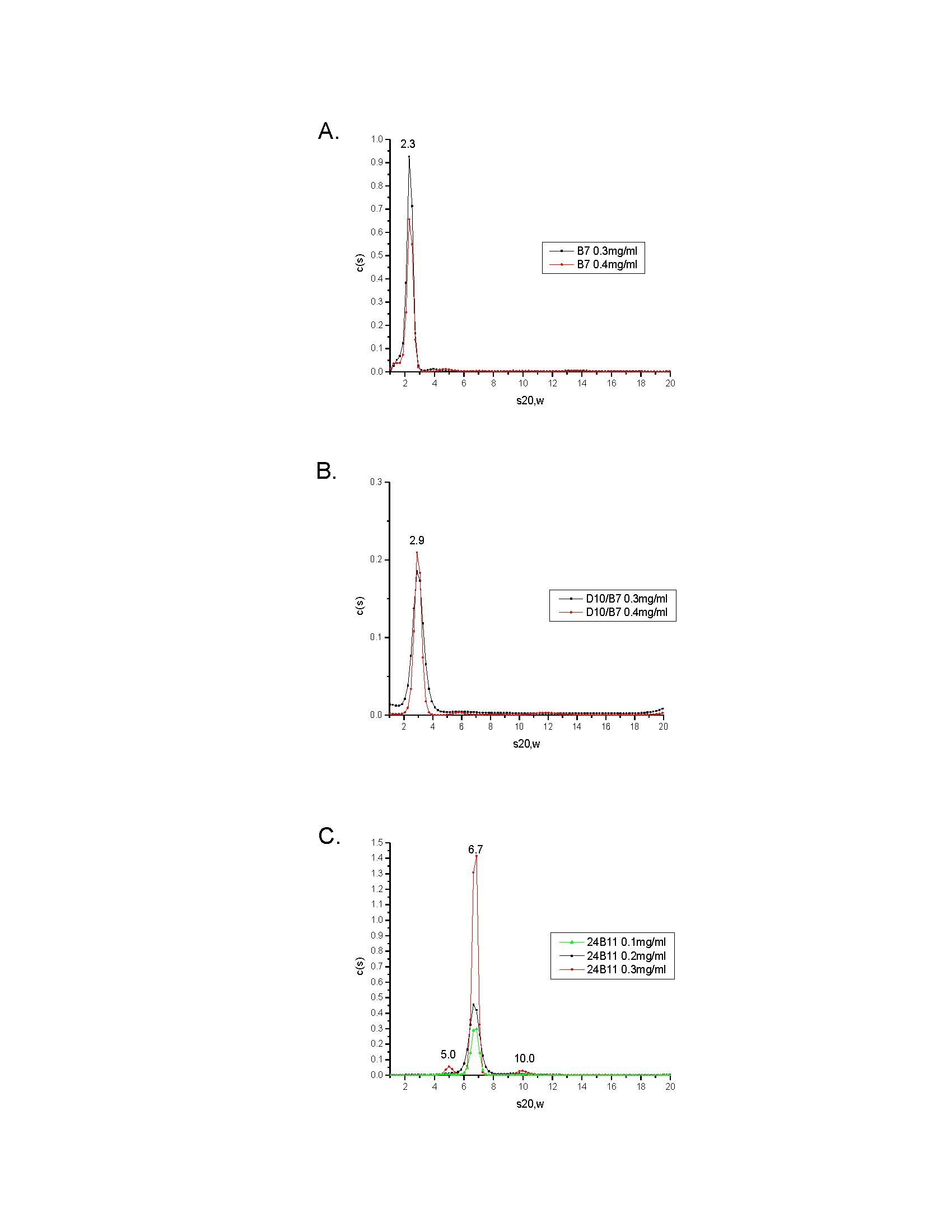

Supplement: Figure S2 — Sedimentation coefficients for 24B11, D10/B7 and RTB-B7. AUC was used to determine sedimentation coefficients for antibodies (A) RTB-B7, (B) D10/B7 and (B) 24B11 at indicated concentrations, as described in the Materials and Methods. For convenience, the corrected sedimentation coefficients (s20,w) are denoted above each sedimentation distribution. (TIFF) [file pone.0099788.s002.tiff]
